# Experiences with healthcare navigation and bias among adult women with sickle cell disease: a qualitative study

**DOI:** 10.1007/s11136-024-03805-x

**Published:** 2024-10-14

**Authors:** Jessica K. Wu, Kyler McVay, Katherine M. Mahoney, Farzana A. Sayani, Andrea H. Roe, Morine Cebert

**Affiliations:** 1 Department of Obstetrics and Gynecology, Penn Medicine, Philadelphia, PA 19104 USA; 2 Perelman School of Medicine, University of Pennsylvania, Philadelphia, PA 19104 USA; 3 Department of Medicine, Division of Hematology and Oncology, Penn Medicine, Philadelphia, PA 19104 USA; 4 Dr. Susan L. Davis, RN, & Richard J. Henley College of Nursing, Sacred Heart University, Fairfield, CT USA

**Keywords:** Sickle cell disease (SCD), Patient experience, Stigma, Bias, Chronic disease

## Abstract

**Purpose:**

The purpose of this study was to use qualitative interviews to understand the experiences of adult women with sickle cell disease (SCD) through daily life and navigating the healthcare system.

**Methods:**

We conducted semi-structured interviews with reproductive-aged women with SCD and performed thematic analysis.

**Results:**

We analyzed interviews from 20 participants. Our data demonstrated three overarching themes: perceptions of disease, transitions of care, and stigma and bias. Participants identified feelings of both empowerment and powerlessness from SCD that evolved over time and globally impacted their lives. The transition from pediatric to adult care was a vulnerable period, both surrounding changes in disease character and challenges transitioning healthcare systems. Finally, participants faced discrimination and prejudice within SCD care, which manifested as disvaluing of their own disease expertise or perpetuation of a “drug-seeking” stereotype. In the context of this bias, some participants prioritized seeking same-race providers.

**Conclusion:**

Experiences with SCD contribute significantly to daily quality of life in women with SCD, and ongoing care gaps exist in relation to their disease. Within our population, SCD as a physical and mental stressor requiring interdisciplinary support should not be underestimated. More robust systems to support the transition from pediatric to adult care are also necessary, both on a healthcare institution level and to support patients’ engagement in their care. Finally, provider education and training on anti-racist practice and both recognizing and eliminating bias are essential to improving care of SCD patients. Possible interactions between sex, gender, and race in the experience of SCD warrant further exploration.

## Abstract (plain language summary)

This study uses patient interviews to describe the experiences of women with sickle cell disease (SCD) navigating the healthcare system. The goal of the study is to highlight patients’ own experiences in order to prioritize patient-centered care in the management of SCD. Our results showed that different individuals perceive living with SCD differently, in both a positive and negative light. The transition from receiving pediatric to adult medical care for their SCD was a vulnerable time period where individuals had to face changes in both their disease and the healthcare system they were navigating. Finally, patients often experienced stigma and biased care from healthcare staff, manifesting as questioning of their own expertise on their disease and perpetuation of “drug-seeking” stereotypes, leading some to value the importance of having same-race providers. These results help better understand how women with SCD experience living with their disease and identify clinical care gaps that need to be filled, such as more robust systems for supporting the transition from pediatric to adult care and increased recognition and directed training on implicit and explicit bias.

## Introduction

Sickle cell disease (SCD) is an inherited blood disorder that affects over 100,000 individuals in the United States [1]. SCD is characterized by acute, severe pain episodes caused by vaso-occlusion, as well as progressive organ damage [2]. With new screening, prevention, and treatment interventions, the mortality rate of SCD in the United States has decreased, with more patients living into adulthood [3]. Despite these advancements, living with SCD continues to be a challenge. Health-related quality of life (HRQOL) has been shown to be significantly worse in SCD patients compared to the general population [4, 5]. Debilitating chronic pain directly contributes to quality of life, with worse HRQOL scores associated with increasing pain [4, 5]. The effects of SCD also impact numerous facets of daily life, including work productivity and mental health, that contribute to overall disease burden [5, 6]. Young adults with SCD must also learn to navigate the healthcare system on their own, with limited support structures for the transition from pediatric to adult care [7, 8].

As with many chronic diseases, patients with SCD interface heavily with the healthcare system. These interactions are frequently perceived negatively by patients and are associated with suboptimal treatment of pain and building of distrust with providers [9, 10]. Patients have also reported unfair treatment in healthcare settings, most often attributed to racial bias or reluctance to provide pain medicine [5]. While the experience of patients being labeled as “difficult” or “drug-seeking” is well-documented, little research exists on the long-term impact of discrimination on individuals with SCD [11, 12].

Women with SCD further represent a vulnerable subpopulation, and limited existing research on this group primarily only focuses on the relation to pregnancy [13, 14]. Reproductive healthcare experiences and perspectives specifically around contraceptive use in women with SCD were the focus of a companion study by our team [15], but more insight is also needed into overall health experiences of these patients. Although the multi-dimensional effects of SCD on daily life and healthcare experiences have been described, existing studies primarily utilize survey data with limited examples of patients’ own voices on their experience. The objective of this study was to use qualitative interviews of reproductive-age women with SCD to better understand these patients’ lived disease experiences, both in their daily lives and in interfacing with the healthcare system.

## Methods

We conducted a descriptive qualitative study between November 2021 and March 2023 using semi-structured interviews from a cohort of reproductive-age females living with SCD. All study procedures were approved by the University of Pennsylvania Institutional Review Board (IRB Protocol #850170). Data collected in this study were also used in a separate study looking at contraceptive attitudes of patients with SCD [15].

Participants were recruited from Penn Medicine hematology clinic advertisements and telephone and e-mail invitations, as well as by electronic study flyers to other US academic hematology centers. Individuals aged 18–45 living with SCD who were assigned female at birth and English-speaking were eligible. Participants were compensated for their time. We obtained university funding to recruit approximately 20 participants, a sample size that has been shown to reliably achieve code saturation [16, 17], though we maintained the option to recruit more participants as needed [18]. We collected participant demographic and medical information, including SCD genotype, which was self-reported and verified in the medical record for patients from our institution. Research assistants hired by the primary investigator were trained by M.C., a researcher with doctoral-level experience in conduct of qualitative research. They conducted semi-structured, open-ended interviews by telephone or an IRB-approved virtual platform using an interview guide developed by A.H.R. and M.C. using literature and clinical expertise. The interview guide was created to elicit participants’ thought processes and experiences around contraceptive use for our companion study, as well as to expand on experiences living with SCD and their relationships with the healthcare system (see Table 1 for example questions). Interviews were recorded using encrypted voice recorders, uploaded on a secured drive, and transcribed verbatim. Transcripts were manually de-identified to promote anonymity and were read by the research team to ensure accuracy. Transcripts were uploaded into NVivo 12 software for coding and analysis.

**Table 1:** Example questions from interview guide for qualitative study of females with SCD in United States hematology centers, 2022

Interview guide domain	Example questions
Background	“Tell me a little bit about yourself.”
Clinical characteristics and healthcare access	“What is a typical week like for you living with SCD?” “Tell me about the medical care you received related to your SCD.”

An initial codebook framework was created deductively from interview guide questions, and additional codes were added inductively after initial review of six (30%) interview transcripts by two independent investigators to account for emerging themes. Two primary coders used the codebook to independently assign codes to five (25%) initial transcripts before conferring with the study team. Investigators met frequently to discuss codebook creation and code assignments for initial transcripts until intercoder reliability with a Kappa statistic > 0.8 was achieved. Investigators and coders met iteratively to discuss code assignments until no new codes were defined, at which point code saturation was assumed. After completion of coding, the team met as a group to develop final themes.

## Results

A total of 20 participants completed interviews. Participants ranged in age from 22 to 45 years (Table 2). All participants were cisgender women. Sixteen participants were patients at Penn Medicine hematology clinics, and four were recruited from other academic institutions. Patient experiences with SCD within our participants reflected three overarching themes: Perception of Disease, Transitions of Care, and Stigma & Bias. Each of the themes and subthemes will be described below. Additional quotations can be found in Table 3.

**Table 2:** Demographic characteristics of participants in a qualitative study of experiences with sickle cell disease among females with SCD in United States hematology centers, 2022 (*N* = 20)

Age (mean, in years)	29.3
Race	
Black or African American	19 (95%)^a^
White	1 (5%)
Obstetric history	
Gravid	9 (44%)
Nulligravid	11 (55%)
Sickle cell disease genotype	
SS	14 (70%)
SC	4 (20%)
SB+	1 (5%)
Unknown	1 (5%)
At least one self-reported vaso-occlusive pain episode in past year	19 (95%)
At least one self-reported hospitalization for vaso-occlusive pain in past year	16 (80%)

a.The racial disparity in percentage of Black vs. white patients in our sample reflects the increased prevalence of SCD among Black individuals, as described in the literature [1]

**Table 3:** Themes and representative quotations describing experiences with SCD among females in United States hematology centers, 2022

Theme	Sub-theme	Representative quotations
Perception of disease	Acceptance and coping	“I try not to think about what I have or—you know, if I’m in pain I try to deal with it. That’s how it works.” (Participant 2) “Every time it grabs you. And that’s no excuse for—to not have a life well-lived. But one must truly understand that the life of a sickle cell patient is hell.” (Participant 18)
	Changing perceptions over time	“And I do know how to manage it now. So I know how to stop the pain attacks and how to maintain it when I do have pain.” (Participant 4) “But as an adult, you have certain responsibilities that you have to tend to.” (Participant 15)
Transitions of care	Disease transitions and logistical challenges	“But once I turned 18, it got way worse.” (Participant 12) “I feel like I had a lot of hospitalizations during that period… Well, stress, obviously, from school, but also probably being away from home, you know, trying to balance coming into adulthood, school.” (Participant 17) “It was pretty difficult to transition to an adult hematologist. I’ve looked before on my own and it was just really hard… I just couldn’t find a good process.” (Participant 16)
	Pediatric versus adult hematologists	“You get so used to being treated a certain way, when you go to adult care, it feels like you were privileged… versus getting adult care because they aren’t nice.” (Participant 7) “So I had the same team, the same care. I was always on the same floor. Everyone was so nice to me. It was always like a family type of thing. And then when I got to adult doctors, everything kind of changed.” (Participant 1).
Stigma and bias	Questioning of patient expertise	“They think you’re being hysterical or exaggerating or not telling the truth, just stuff like that. They don’t really care.” (Participant 7) “There was one time when some ER doctors, they just were not listening to anything that I said… It gets very frustrating and infuriating when I’m telling them something and they’re not listening to me.” (Participant 15)
	“Drug-seeking” stereotypes	“We’re a burden. We’re a bother. We’re junkies who are annoying and won’t shut up about it… We’re a nuisance… Because we’re in pain, we’re pressing the button in the hospital room… And I know exactly what I need, but I don’t look sick, so it just looks like I’m coming in here to have a good time, and there’s no good time to be had. Whatever you think, we’re drug seeking and looking for this and out for that, you don’t even know. They have no idea what’s behind the curtain of sickle cell.” (Participant 18)
	Desire for same-race providers	“And so those were my biggest factors, and so though sickle cell was a big factor, I think finding a Black woman was the biggest factor.” (Participant 19)

### Perception of disease

#### Acceptance and coping

Among our participants, living with SCD was a source of feelings of both powerlessness and empowerment. One participant described her reliance on the healthcare system: “For every problem in my life, I have been instructed to take this or listen to that or this doctor, or there’s this medical treatment… Everything is outside of myself” (Participant 18). Others spoke to how their disease made them feel isolated, especially during childhood: “I have to deal with this my whole life… That gave me the realization on like, I couldn’t do other things like the other kids could” (Participant 6).

Participants also spoke to their acceptance of their disease and learning to adapt their lives around SCD: “It was hard, but I guess growing up with it, it just kind of becomes part of your life” (Participant 4). Finding a sense of normalcy was a common coping strategy—participants sought to adjust to their “new normal” and continue with their daily routines.

Despite attempts at coping and acceptance, SCD continued to have negative effects on social and professional lives. Some saw an impact on their relationships and others’ perceptions of them: “Who would want to hang out with someone who couldn’t keep up, who always had to stop to catch their breath” (Participant 11). Work was also affected, with some participants losing jobs due to their SCD: “I think sickle cell will, of course, impact every job that I have because it’s kind of unpredictable” (Participant 8). More broadly, SCD was felt to be a major stressor on mental health. One participant described the energy needed to live with the disease: “Sickle cell is, at this point, my full-time job… It works me, and it’s tiring, and it’s frustrating” (Participant 18). Living in constant pain from SCD also impacted mental health: “When you’re always in pain, your mind goes to dark places” (Participant 18).

While most individuals described SCD as having a negative impact on mental health, one individual felt empowered by living with SCD: “I would pick sickle cell over anything. This makes me strong… Sickle cell is my superpower, it built me this way” (Participant 1). Statements like these showcase the psychological resilience of many individuals with SCD, especially as their lived experience and comfort with their disease accumulates over time.

#### Changing perceptions over time

Participants’ perceptions and experiences with their disease were reported to evolve with time and age. Many spoke to how they grew into their disease as they learned how to manage it: “Now it’s becoming more bearable. I’m understanding it more, I can take care of myself more” (Participant 2). As participants developed familiarity with their symptoms, they were often able to predict their trajectory and intervene early “to not let it get past a certain point” (Participant 8), allowing them to have more control. Participants were also able to find ways to integrate SCD with their lives, including requesting accommodations for school exams or confiding in co-workers.

### Transitions of care

#### Disease transitions and logistical challenges

Another prominent theme in our interviews was surrounding the transition from pediatric to adult care. For some, their disease worsened with the stressors of adulthood: “When I went to college all that changed. I was always in a hospital… And I think it was because of stress… because I moved away” (Participant 8). Many participants also felt that they needed earlier preparation for physical changes to expect with their disease, with some describing not fully understanding what was happening in their body as they faced dealing with their disease on their own: “I didn’t understand sickle cell fully. And even my pediatric hematologists, they never really explained it to me… I never understood what was going on inside my body” (Participant 3). With this came a feeling of inadequate preparation for how their disease would change with age: “I wish that my doctors would have warned my parents, like, she should look out for this or y’all should be aware of this—because with age and with change, sickle cell will show up differently” (Participant 8).

The logistics of transitioning from pediatric to adult care also proved challenging for many, including finding the right adult hematologist: “I feel like I lost so many years of my life because I was in this twilight zone of doctors and jumping everywhere trying to find the right doctor” (Participant 3). Others commented on challenges with moving toward independent insurance, as well as lack of information transfer and care continuity when switching providers.

#### Pediatric vs. adult hematology care

Finally, specific experiences with pediatric versus adult hematologists often differed. Some felt that the difference in closeness of care was a prominent adjustment: “You don’t have that constant oversight with an adult hematologist that you do with a pediatric hematologist” (Participant 13). Others found adult hematologists less approachable: “It’s scary as an adult. I shouldn’t be scared to ask questions about my health and be scared to ask about my care, but I know some doctors just… don’t want to explain” (Participant 9). As adults, participants sometimes felt like they received less care or their concerns were taken less seriously: “It’s terrible because the care is not emphasized. Once you become an adult, it’s like, oh, you’re an adult, you got this. But as a child, of course, children are going to get more care. They’re going to listen to them more… But as an adult, a lot of the times… they see it as a fabrication” (Participant 8).

### Stigma and bias

#### Questioning of patient expertise

When navigating healthcare settings, participants described facing discrimination and prejudice when seeking care for their SCD. Their symptoms and expertise about their disease were frequently questioned by providers: “You have a whole bunch of doctors telling you that you aren’t sick because you look fine or you can tolerate the pain” (Participant 1). Primarily around pain, participants felt dismissed by providers who they felt were inadequately trained to care for SCD: “I’ve had doctors and nurses completely disregard my pain. I’ve had nurses tell me that they’re not going to give me any more medicine. I’ve had a nurse and a doctor actually ask me if I was in as much pain as I claim I am. I’ve had experiences where people are questioning the reason why I’m even here… Looking for that experience within a doctor or within a nurse… I’m looking for the care that I deserve” (Participant 13). Some felt that providers had preconceived notions about patients with SCD: “They kind of have this wall up, or they have this thing up that all sickle cell patients are fit into one box” (Participant 8).

#### “Drug-seeking” stereotypes

A specific judgment from providers that some participants described was the stereotype of SCD patients being “drug-seeking”: “People with sickle cell disease are stigmatized as being drug addicts and they’re only coming in because they want a fix or they want medical-grade medicine to get high” (Participant 13). They sometimes felt that their familiarity with their symptoms and treatment was instead viewed as a component of this stereotype: “Having an understanding of your illness and what works for you… instead of doctors or physicians looking at it as a positive theme, to them it’s more like a red flag. And so, instead of them thinking, okay, this person knows what they need, they think I’m just seeking out medication, like I’m an addict to pain meds” (Participant 15). One participant also spoke to tension with her doctor around long-term opioid use for her SCD: “It’s like a lot of doctors… are like, you have sickle cell, you’re going to be in pain, you might as well get comfortable with it now, you’re going to be on narcotics for the rest of your life… That’s not the way it should be. And I feel like I was just dismissed” (Participant 3).

#### Desire for same-race providers

Finally, a few participants commented on their prioritization of finding same-race providers, whether for SCD or for other specialty care. One participant stated that “finding a Black woman was the biggest factor” in choosing a provider. Race served as one way for patients to feel more comfortable in their touchpoints with the healthcare system. “I was looking for a Black woman who would be my gynecologist… I was really interested in her feedback and her opinion” (Participant 19). Others felt that Black providers had more familiarity with SCD, with one participant describing her town’s providers as inexperienced in SCD: “There’s not a big Black demographic, so there’s not a huge sickle cell demographic. So, me going there, they wanted to learn… But you’re hindering me… because you want to learn” (Participant 15). Others simply wanted to see their racial identity represented in their care team: “I have the understanding of needing to see yourself in… your providers and having people that are in your corner. I think that was my biggest thing on my list” (Participant 19).

## Discussion

This study used qualitative interviews to analyze patient experiences with SCD and the medical system among reproductive-age women with SCD in the United States. The three overarching themes we found in our data address SCD quality-of-life experiences that did not explicitly relate to sex or gender: perceptions of disease, transitions of care, and stigma and bias. Participants described SCD as a significant stressor on personal lives and mental health, as well as a source of both powerlessness and empowerment. The transition from childhood to adulthood with SCD was universally challenging, both in regards to physical changes and experiences with the healthcare system. Finally, participants frequently faced stigma and bias as their experiences with pain were disregarded and used to fuel negative stereotypes.

### Perceptions of disease

Among our study participants, living with SCD was a source of feelings of both powerlessness and empowerment, influencing how they coped with their disease and navigated work, friends, and relationships. This aligns with previous literature—in the international Sickle Cell World Assessment Survey, researchers found that SCD had a major impact on education, employment, and emotional well-being [19]. Moreover, our findings build on a prior study that found that participants living with SCD experienced challenges with accepting their diagnosis, a process that occurred from childhood well into adulthood [20]. Another study found that people living with SCD experience extraordinary stressors while trying to adapt to basic responsibilities of living, such as ability to meet basic needs, financial strains, and disruptions in family and friendships due to disease burden [21]. Our study highlights the continued need to support the emotional, social, and financial well-being of women living with SCD. These factors are known to influence quality of life and the holistic burden of SCD.

### Transitions of care

Participants in our study discussed multi-dimensional challenges surrounding the transition from pediatric to adult care for SCD. Transitions of care are a well-established period of vulnerability in medical care. Existing barriers to effective transitions primarily include lack of guidance on identifying adult institutions and providers specializing in SCD [22]. Transition pathways are variable and can depend on whether relationships exist between pediatric and adult hospitals within a given instutition. Other barriers may be more individualized, resulting from patients’ prior negative experiences within the healthcare system, socioeconomic factors, and developmental maturity and skills [23]. Feelings expressed by participants in our study echo those identified in the literature—adolescents frequently acknowledge the importance of this transition but feel poorly prepared to transition to adult-oriented care [22], which has been shown to have significant health consequences. SCD itself has already proved to be a stressor across multiple life domains, including in work and mental health, as reiterated in our study. SCD pain has been shown to impact productivity at work, absenteeism, and unemployment [24], which in turn can exacerbate pain frequency [25]. Psychological effects of SCD are also well documented, with significantly higher rates of depression compared to the general population (26% vs. 9.5%) that often goes untreated [26], which also cyclically impacts worse pain outcomes and hospital admissions [25, 27, 28].

Compounding these underlying stressors, the greatest acute care utilization and rehospitalization rates for SCD have been demonstrated in patients age 18–30, as young adults are at risk both as their disease worsens and they transition between care systems during this vulnerable period [29, 30]. In one study looking at long-term follow-up for patients from the Dallas Newborn Cohort, young adults transitioning from pediatric to adult care were at high risk of early death, especially soon after this transition [30]. Another recent study identified that individuals with SCD who experience a delay in transitioning from pediatric to adult care of > 6 months were twice as likely to be hospitalized compared to those who transitioned within 2 months [31]. Young adults are therefore an especially vulnerable population with a high risk of mortality at the interface between pediatric and adult medical care [30].

These themes identified in our study build on prior literature highlighting the importance of supporting three phases of the care transition process: future orientation, preparation, and time of transition [32]. More resources need to be put in place to help young adults take ownership of their care and practice self-advocacy and self-management [32, 33]. Part of this includes ensuring patients are well educated on their disease while still in pediatric care to start the preparation process early. Providers should also be encouraged to support patients as experts in their own bodies, especially as they transition to adulthood—when providers are proactive and engage patients and families in their healthcare, patients may feel more empowered [33]. Positive relationships between patients and their providers have also been shown to be a facilitator for effective transition [23]. Streamlined systems for handing off patients from pediatric to adult providers during the time of actual transfer, particularly within the same or partner institutions, should also be implemented, as our study participants struggled with both logistical challenges of finding new providers and the unfamiliarity of losing long-term pediatric teams.

### Stigma and bias

When navigating healthcare settings, our study participants described facing discrimination and prejudice when seeking care for SCD. Stigma is a well-researched experience that impacts those with SCD, but these experiences have not been well described by patients themselves in a US context. Most relevant literature speaks to international experiences—in one study of adults with SCD in Brazil, stigma was a multidimensional experience that posed challenges with the general public, family members, and healthcare workers [34]. Another study conducted in Ghana highlighted young adults with SCD’s experiences of exclusion and the impact on health outcomes [35]. Patients with SCD have long been impacted by years of structural racism and the ensuing prejudices and biases [36, 37]. Stigma associated with SCD has been shown to have wide-ranging consequences, including effects on psychological and physiological well-being, social relationships, and care-seeking behaviors [12]. Challenges in receiving adequate care and stress associated with racial stigma can lead patients to avoid care altogether, further increasing the risk of life-threatening complications [37]. Perceived stigma also has a negative relationship with self-efficacy, as repeated discrimination diminishes ability to engage in disease-related self-efficacy and self-care, leading to poorer health outcomes [38]. While stigmatization may begin in childhood, it is often most evident as young adults transition from pediatric to adult care, adding to an already vulnerable period [39].

The feelings of exclusion identified in international patient cohorts were corroborated by our data—participants felt “othered” both by providers disbelieving and stereotyping them, as well as by racial dissonance between them and their providers. One participant who identified as Black noted that finding a Black provider was one of her top priorities in seeking care in order to “see [herself]” in her providers, an idea that has not been explicitly expressed in the SCD literature by patient voices. The role of anti-racism efforts in this realm cannot be underestimated, as increased education on racist stereotypes and implicit bias against SCD patients is critically needed. Providers should not only be educated about systemic biases, but also trained on ways in which to dismantle harmful stereotypes, such as the “drug-seeking” stereotype—where behaviors driven by genuine pain symptoms in an attempt to control pain are presumed to be symptoms of opioid addiction, a misperception that results in problematic and inadequate pain management [40]. Given that our study included an exclusively female cohort, one participant also prioritized having a female provider, speaking to gender as another area of potential bias that requires future study. The burden of maximizing quality of care for SCD needs to be shifted from patients striving to be “good patients” toward what it means to be a “good provider” for these patients, a potential area for future qualitative study.

### Strengths and limitations

The primary strength of this study is that it represents one of the first studies to describe the lived experience of women with SCD in patients’ own words, reflecting on themes that are both widely acknowledged, such as stigma surrounding SCD, as well as those not previously described, including positive elements of the disease. Two of our participants described SCD as a source of empowerment and strength, with one calling SCD her “superpower.” Through open-ended questions about disease experiences, we were also able to elicit patients’ greatest challenges navigating SCD. These themes help direct the need for further qualitative study about unexplored aspects of the SCD experience.

The primary limitation of this study is its small sample size, therefore providing a limited view of our population’s experiences. However, though small, our sample was recruited from multiple academic medical centers, allowing us to draw from experiences from a diversity of care locations to identify critical themes that can be expanded on in larger cohorts. Another limitation is that because our sample only included reproductive-age women with SCD, which was driven by our goal to not only examine overall health experiences but also reproductive healthcare experiences around contraceptive use in a companion study, our interviews were unable to describe differences in SCD experiences related to gender or sex. Though gender- or sex-related components of participants’ SCD experiences were not prominently featured in our data, disparate experiences between women and men with SCD cannot be excluded, and further studies should explore this comparison by interviewing female and male SCD patients.

## Conclusion

This study used qualitative interviews to explore experiences of reproductive-age women with SCD and the disease impact on their quality of life, as well as to examine ongoing care gaps for these patients. Robust interdisciplinary support systems are needed to manage SCD as a physical and mental stressor, especially during transitions of care. Providers also need to prioritize recognizing and counteracting bias in care of SCD patients.
